# A Fast And Versatile Method for Simultaneous HCR, Immunohistochemistry And Edu Labeling (SHInE)

**DOI:** 10.1093/icb/icad007

**Published:** 2023-03-02

**Authors:** Aida Ćorić, Alexander W Stockinger, Petra Schaffer, Dunja Rokvić, Kristin Tessmar-Raible, Florian Raible

**Affiliations:** Max Perutz Labs, University of Vienna, Vienna BioCenter, Dr. Bohr-Gasse 9/4, 1030, Vienna, Austria; Research Platform “Rhythms of Life,” University of Vienna, Vienna BioCenter, Dr. Bohr-Gasse 9/4, A-1030, Vienna, Austria; Max Perutz Labs, University of Vienna, Vienna BioCenter, Dr. Bohr-Gasse 9/4, 1030, Vienna, Austria; Research Platform “Rhythms of Life,” University of Vienna, Vienna BioCenter, Dr. Bohr-Gasse 9/4, A-1030, Vienna, Austria; Research Platform “Single-Cell Regulation of Stem Cells,” University of Vienna, Vienna BioCenter, Dr. Bohr-Gasse 9/4, A-1030, Vienna, Austria; Max Perutz Labs, University of Vienna, Vienna BioCenter, Dr. Bohr-Gasse 9/4, 1030, Vienna, Austria; Research Platform “Rhythms of Life,” University of Vienna, Vienna BioCenter, Dr. Bohr-Gasse 9/4, A-1030, Vienna, Austria; Max Perutz Labs, University of Vienna, Vienna BioCenter, Dr. Bohr-Gasse 9/4, 1030, Vienna, Austria; Research Platform “Rhythms of Life,” University of Vienna, Vienna BioCenter, Dr. Bohr-Gasse 9/4, A-1030, Vienna, Austria; Max Perutz Labs, University of Vienna, Vienna BioCenter, Dr. Bohr-Gasse 9/4, 1030, Vienna, Austria; Research Platform “Rhythms of Life,” University of Vienna, Vienna BioCenter, Dr. Bohr-Gasse 9/4, A-1030, Vienna, Austria; Alfred Wegener Institute, Helmholtz Centre for Polar and Marine Research, Am Handelshafen 12, 27570 Bremerhaven, Germany; Carl-von-Ossietzky University, Carl-von-Ossietzky-Straße 9-11, 26111 Oldenburg, Germany; Max Perutz Labs, University of Vienna, Vienna BioCenter, Dr. Bohr-Gasse 9/4, 1030, Vienna, Austria; Research Platform “Rhythms of Life,” University of Vienna, Vienna BioCenter, Dr. Bohr-Gasse 9/4, A-1030, Vienna, Austria; Research Platform “Single-Cell Regulation of Stem Cells,” University of Vienna, Vienna BioCenter, Dr. Bohr-Gasse 9/4, A-1030, Vienna, Austria

## Abstract

Access to newer, fast, and cheap sequencing techniques, particularly on the single-cell level, have made transcriptomic data of tissues or single cells accessible to many researchers. As a consequence, there is an increased need for *in situ* visualization of gene expression or encoded proteins to validate, localize, or help interpret such sequencing data, as well as put them in context with cellular proliferation. A particular challenge for labeling and imaging transcripts are complex tissues that are often opaque and/or pigmented, preventing easy visual inspection. Here, we introduce a versatile protocol that combines *in situ* hybridization chain reaction, immunohistochemistry, and proliferative cell labeling using 5-ethynyl-2′-deoxyuridine, and demonstrate its compatibility with tissue clearing. As a proof-of-concept, we show that our protocol allows for the parallel analysis of cell proliferation, gene expression, and protein localization in bristleworm heads and trunks.

## Introduction

Advancements in sequencing techniques have significantly increased the number of organisms for which molecular research can be conducted in. Moreover, single-cell RNA sequencing allows such investigations on the level of individual cell types (Fonseca et al. 2016; Stark et al. 2019). In turn, these high throughput methods have generated a need for validating digital expression data in the corresponding organism, specifically for the visualization of gene-expression on the three-dimensional level.

In recent years, strategies complementary to traditional enzyme-coupled *in situ* hybridization (ISH) have been developed. One of them is *in situ* hybridization chain reaction (HCR). *In situ* HCR is faster to perform than traditional ISH, and works at lower temperatures, which aids maintaining tissue integrity. Moreover, *in situ* HCR can be multiplexed by using different fluorophores compatible with fluorescent imaging (Choi et al. 2016), making *in situ* HCR an attractive companion technique to single-cell sequencing.

For *in situ* HCR, short DNA probes are synthesized that are complementary to the target transcript. These probes can be designed using web-tools and ordered on bulk scale, similar to primers, which makes production fast and affordable. Amplifier oligonucleotides carrying fluorophores are then annealed to an overhang region on the probes, triggering the release of a hairpin structure, and thereby revealing the binding sequence for another amplifier molecule. This leads to a chain reaction of linear signal amplification. The latest version of the method, termed HCR 3.0 (Choi et al. 2018), employs split initiator sequences: Two DNA probes form a probe pair and have to successfully bind next to each other to form a shared initiator sequence. Off-target binding or trapping of individual probes therefore should be less likely to cause signal, improving sensitivity of the method.

Whereas, high sensitivity is an important feature for the use of *in situ* HCR to validate scRNAseq data, there are also challenges and open questions to the use of this technique: First, the acquisition of images from *in situ* HCR-stained samples by confocal microscopy bears potential problems in larger specimens, as opaqueness of tissue or pigmentation are known to prevent deep imaging (Tainaka et al. 2015). While various tissue clearing methods have been developed to overcome imaging problems in such specimens (Vieites-Prado and Renier, 2021), their compatibility with RNA detection has not been systematically assessed.

Second, while visualizing RNA is a relevant experimental aim, there are contexts where covisualization of proteins by immunohistochemistry (IHC) would yield additional insights. For example, such codetection could help to benchmark gene expression in cells or tissues for which protein markers are already established, or to directly assess ratios of RNA and corresponding protein (Darmanis et al. 2015; Albayrak et al. 2016). Combining the visualization of protein and RNA can therefore be used to study developmental processes or gene regulation in ways each technology on its own would not allow.

Few protocols have been published that combine the convenience, flexibility, and sensitivity of *in situ* HCR with immunostainings. Those describing a combined approach either rely on the availability of commercial antibodies and proprietary technology (Schwarzkopf et al. 2021) or follow a longer protocol that performs immunostaining after finishing *in situ* HCR (Ibarra-García-Padilla et al. 2021; Elagoz et al. 2022), which delays workflows and potentially lowers the achieved signal.

**Fig. 1:**
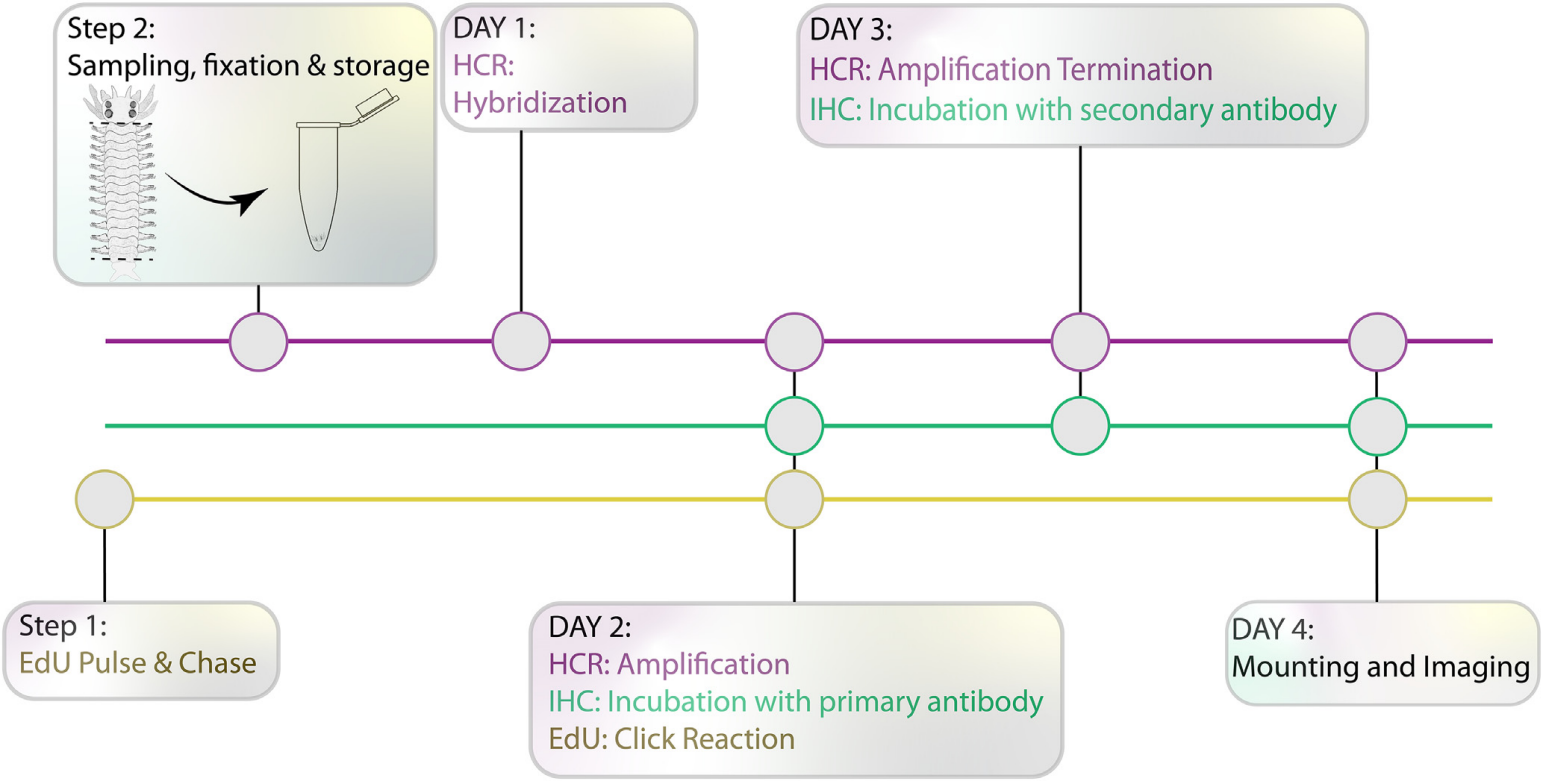
Experimental workflow for simultaneous *in situ* HCR, IHC, and EdU labeling. Following the initial treatment with EdU, animals are sampled and fixed for *in situ* HCR. Our protocol allows for the implementation of HCR amplification, EdU detection (click reaction), and incubation with primary antibody for IHC on day 2, resulting in a total of 4 days from sampling to imaging.

Here we present a protocol (termed Simultaneous HCR, IHC, and EdU labeling/SHInE; Fig. 1) that addresses both of these challenges: SHInE combines *in situ* HCR with IHC, allowing codetection of RNA and protein. By introducing the primary antibody incubation of the IHC simultaneously to the amplification step of *in situ* HCR, SHInE saves experimental time and keeps the number of washing steps following amplification to a minimum. Moreover, SHInE is compatible with tissue clearing using the recently developed DEEP-Clear method (Pende et al. 2020), extending the portfolio of labeling techniques for this method.

Our protocol also offers the flexibility of using non-commercial, self-designed HCR probes along with custom antibodies and home-made buffers. In addition, SHInE has been adapted to include additional molecular assays, such as the labeling of proliferating cells with 5-ethynyl-2’-deoxyuridine (EdU) (Salic and Mitchison, 2008; Zattara and Özpolat, 2020). This further enables the study of complex biological processes.

To demonstrate the functionality of our protocol, we provide results for different sets of HCR probes, different antibodies, and in different tissues of the marine bristle worm *Platynereis dumerilii*. This invertebrate has gained importance as a model organism in various biological fields, including evolution, development, regeneration, neurobiology, reproduction, chronobiology, and ecology [reviewed in (Özpolat et al. 2021)]. As in other model systems, conventional, riboprobe-based ISH has been successfully established for gene expression studies in this species (Tessmar-Raible et al. 2005). This approach has recently been expanded to the use of single-color and multiplexed HCR 3.0 (Revilla-i-Domingo et al. 2021; Kuehn et al. 2022). By combining HCR labeling of RNA with IHC, EdU, and tissue clearing, SHInE provides a new and flexible tool for studying a broad range of biological processes in *P. dumerilii*, and can likely be adapted to other species.

## Materials and methods

A schematized workflow of the SHInE protocol is presented in Fig. 1. A detailed version of the protocol, including all reagents and buffers, has been deposited on protocols.io (DOI: dx.doi.org/10.17504/protocols.io.5qpvobnyzl4o/v1). This version contains detailed steps with estimated time requirements, pause points, and steps that can be adjusted according to specific experimental needs (Supplementary File 1).

### Animal husbandry

*Platynereis dumerilii* worms were kept in continuous culture at the Max Perutz Labs Vienna Marine Facility under a 16:8 light: dark regime. For details on animal culture, see (Hauenschild and Fischer, 1969; Kuehn et al. 2019). In short, adult worms were mated, and batches were reared in flat, transparent plastic containers. Depending on batch size, around 50 to 200 animals can be kept together this way, in volumes ranging from 0.5 to 1 l of sea water mix.

Animals were kept in a 1:1 mixture of sterile-filtered natural sea water and artificial sea water (Tropic Marin Classic) adjusted for salinity (to 34–35 parts per thousand).This mix is referred to as ASW/NSW throughout the manuscript. Animals were fed twice a week, once with organic spinach leaves cut to roughly 3 × 3  mm pieces in a blender, and once with a mix of ground Tetramin fish food and Spirulina powder.

### Posterior amputations

Sibling worms aged 3–5 months and of a size between 40 and 50 segments were sampled. Trunk pieces were surgically amputated by anesthetizing animals in 7.5% MgCl_2_ mixed 1:1 with ASW/NSW, then removing all segments posterior to the 30^th^ segment with a scalpel, cutting perpendicular to the animals’ body axis, similar to previous reports (Planques et al. 2018). Amputated animals were then placed in fresh ASW/NSW, after which they were left for recovery and regeneration in a clean culture box of 0.5l for up to 30 animals. Animals used for posterior amputation experiments were not used for any additional experiments.

For each experimental condition, 3–5 animals were used. Representative samples were selected and imaged for figures of this manuscript. Statistics in Fig. S1 and Fig. S2 are based on evaluation of multiple biological samples.

### Sampling of worm heads for *in situ* HCR

Wild-type worms of similar age and developmental stage were sampled at zeitgeber time (ZT) 22.

### EdU pulse labeling and click reaction

The concept of EdU incorporation (Salic and Mitchison, 2008; Zattara and Özpolat, 2020) was used to label proliferating cells in the animals. To this end, worms were transferred to glass beakers and incubated with 10 µM EdU in ASW/NSW.

For EdU labeling worm heads, animals were incubated with EdU at the onset of darkness (ZT16) for 6 hr. Worm heads were subsequently sampled at ZT22.

For EdU labeling of worm blastemas, animals that underwent amputations (see above) were incubated for 1h.

The “click” reaction is the detection step during which fluorescent dyes are attached to EdU to label those cells that previously incorporated EdU during DNA synthesis. The reaction was performed according to the manufacturer’s protocol using the Click-iT™ EdU Cell Proliferation Kit for Imaging, with Alexa Fluor™ 488 dye. In short, following HCR probe binding and washes, samples were incubated for 30 min with Alexa Dye azides to label EdU in a copper-catalyzed, enzyme-free stable reaction. For more details, refer to the manufacturer’s protocol or the SHINE protocol, day 3, Step Case “HCR and EDU” or “HCR, EDU, and IHC,” Step 12.

### Microscopy parameters and image processing

**Fig. 2:**
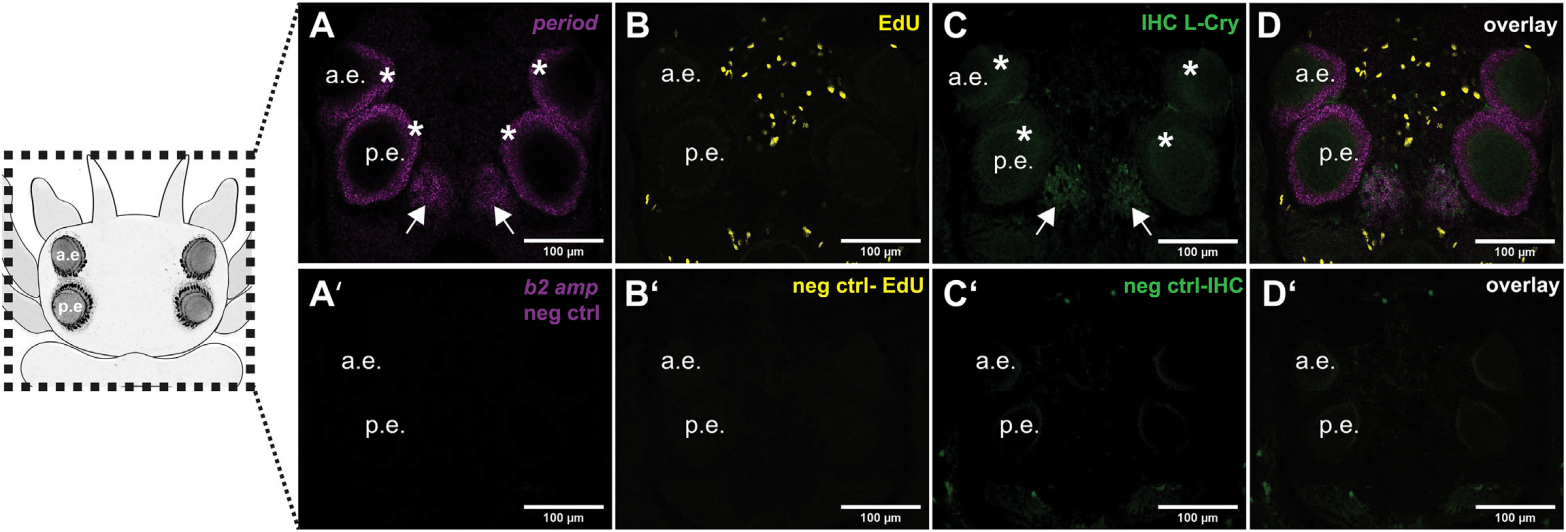
Codetection of HCR probes, immunolabels and EdU in *Platynereis* heads. Head samples (schematized on the left) were processed according to the SHInE protocol and imaged on a confocal microscope. (A and A’) Detection of *Platynereis period* mRNA by *in situ* HCR (A) shows staining in both pairs of eyes and in an oval-shaped domain of the posterior brain. These are absent in negative controls (A’); (B and B’) Detection of proliferating cells by EdU incorporation and visualization (B) reveals sparse proliferation of cells throughout the worm head, but not in the eyes or the oval-shaped posterior domain. No staining is observed in the respective negative control (B’); (C and C’) Immunodetection of L-Cry, a photoreceptor protein, reveals its presence in the eyes and the oval-shaped posterior domain (C). (D and D’) The overlay of all three labels (D) shows strong colocalization of L-Cry and period, while the respective negative controls (D’) exhibit very low background signal. a.e.: anterior eye and p.e.: posterior eye.

**Fig. 3:**
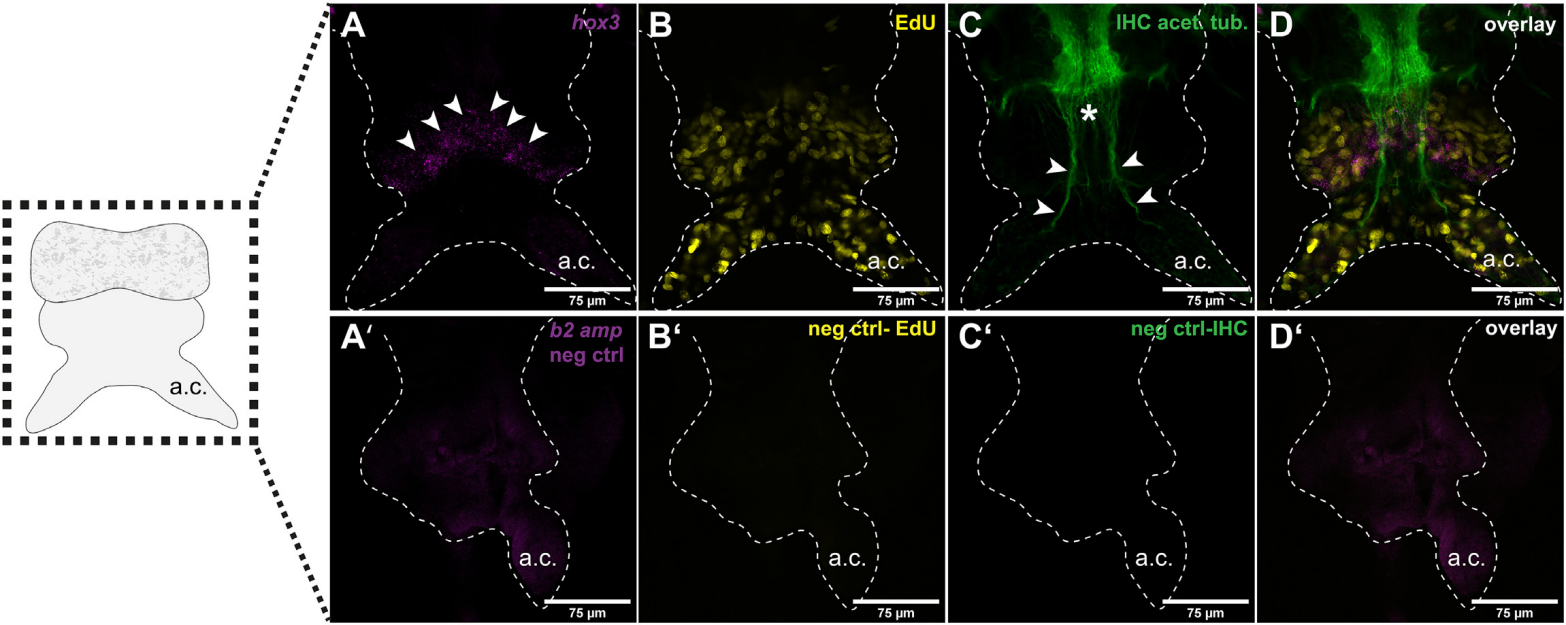
Codetection of HCR probes, immunolabels and EdU in posterior regenerates. Samples of posterior regenerating tissue at three days post caudal amputation (schematized on the left) were processed according to the SHInE protocol and imaged on a confocal microscope. (A) Regenerates exhibit a well-defined area of *hox3* expression, referred to as segment addition zone (demarcated by arrowheads); (B) Visualization of proliferating cells by EdU incorporation and detection reveals many proliferating cells throughout the regenerating tissue, especially in the anal cirri and the area surrounding the segment addition zone; (C) Immunodetection of acetylated tubulin highlights the ventral nerve cord (asterisk) and its projections (arrowheads) into the anal cirri (arrowheads) (C). (D) Overlay of all channels; (A’–D’) As in Fig. 2, the respective negative controls exhibit little to no background signal; autofluorescence in glandular tissue is observed in the HCR negative control (A’), but lacks the granularity and intensity of HCR signal and can therefore be easily distinguished; a.c.: anal cirrus.

**Fig. 4:**
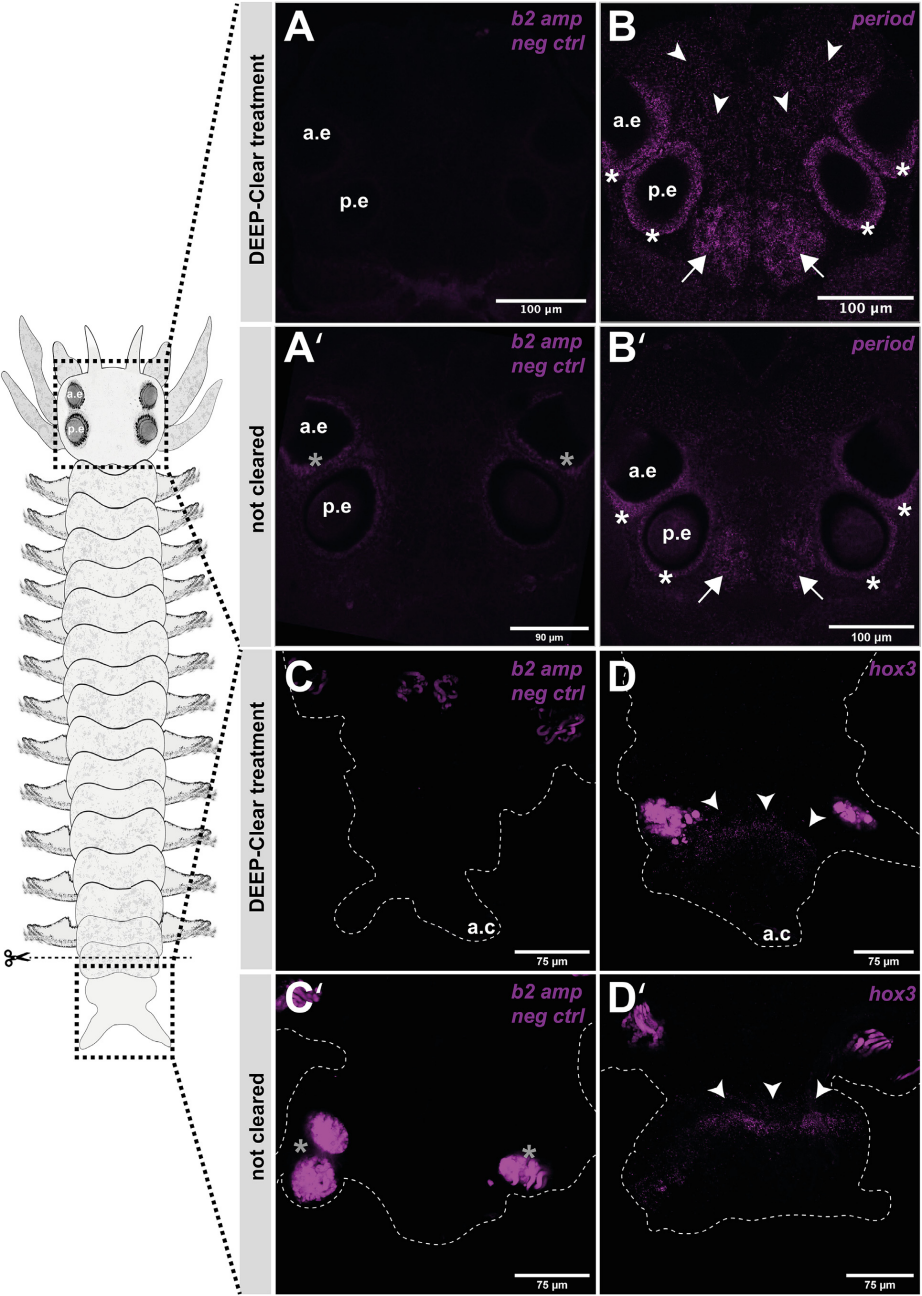
Compatibility of HCR detection with tissue clearing. *Platynereis* heads show differences in background signal in cleared vs. not-cleared individuals. (A and A’) Autofluorescence can be seen around and in the eyes in the negative control for untreated samples (asterisks in A’), while there is no such signal in the negative control of cleared heads (A’). (B, and B’) *Pladu-period* signal is strong around the eyes and in the brain nuclei of the posterior medial brain in *P.dumerilii*. Stronger HCR intensity is observed following tissue clearing (B), which highlights additional, less centralized signal *of Pladu-period*. (C–D’) HCR signal intensity is less affected by clearing in *Platynereis* blastema samples. Negative controls (C and C’) reveal autofluorescence in glandular structures in *Platynereis* trunks as observed previously (Hasse et al. 2010; Asadulina et al. 2012), and is still visible after tissue clearing. (C–C’). *Pladu-hox3* signal is clearly defined and restricted to the segment addition zone (D–D’, arrowheads). Enhancement of signal intensity is not observed following tissue clearing.

Images were taken on a Zeiss LSM 700 inverse confocal microscope using Plan-Apochromat 20x/0.8, WD 0.55 mm and LD LCI Plan-Apochromat 25x/0.8 mm (oil immersion) lenses. Upon screening, the acquisition parameters were set according to the specimen with the strongest fluorescence and applied on one entire set of samples, thereby avoiding possible overexposure and ensuring comparability. Using FIJI (Schindelin et al. 2012), regions of interest (ROIs) were selected on every raw image and fluorescence intensity was determined by multiplying the measured mean gray value of a ROI with its corresponding area. Signal-to-noise ratio was calculated by dividing the fluorescence intensity of each sample by the average fluorescence intensity of the corresponding controls. Contrast (a linear adjustment) was enhanced in FIJI (Schindelin et al. 2012) for every channel separately. Equal settings were used within each experiment to ensure that samples could be quantitatively compared. Minimum and maximum displayed fluorescence values were set as follows: Fig. 2 and Fig. 3: 339–2465 for IHC (Cy3) channel and 0–2372 for EdU (AF488) channel; Fig. 4: 482–808 for HCR (B2 Amplifier—Alexa 647); Fig. S1:1237–2569 for HCR (B1 Amplifier—Alexa 546). Panels were arranged in Adobe Illustrator.

### HCR Probes

HCR probes were designed against the listed target genes (Table 1) using a probe maker tool developed by Ryan Null (Kuehn et al. 2022) that was installed using Python v3.9.2 and jupyterlab 3.0.10.

**Table 1:** Target genes, amplifiers and used fluorophores for the HCR probes reported in this study.

*Gene*	Reference/source	Amplifier	Fluorophore
*Pladu_hox3*	GenBank JQ424894.1	B2	Alexa 647
*Pladu_pdp1*	this study (GenBank OQ450185)	B1	Alexa 546
*Pladu_per*	this study (GenBank OQ450184)	B2	Alexa 647

Respective probe sequences can be found in Supplementary File 2. B2-coupled HCR probes directed against an unrelated sponge gene were provided by Dr. Roger Revilla-i-Domingo (University of Vienna) as negative controls.

The number of probe pairs depended on the length of each target sequence. As detailed in Supplementary File 2, we used 30 probe pairs for *Platynereis hox3*, 49 probe pairs for *Platynereis pdp1*, and 27 probe pairs for *Platynereis per*.

### Antibodies

Details on the used antibodies are listen in Table 2.

**Table 2:** Target proteins and specifications of the antibodies used in this study.

Target	Manufacturer/source	Type	Species of origin
Acetylated alpha-Tubulin	Sigma-Aldrich, #T6793	Monoclonal	Mouse
*Pladu*-L-Cry	Monoclonal Antibody Facility at Max Perutz Labs; (Poehn et al. 2022; Zurl et al. 2022)	Monoclonal	Mouse
anti-Mouse IgG (H + L) Cross-Adsorbed Secondary Antibody, Cyanine3	Invitrogen, #A10521	Polyclonal	Goat

## Results

### SHInE allows codetection of HCR probes, protein, and nuclear labels in whole-mount samples

To address the ability of SHInE to covisualize RNA, protein and S-phase-labeled nuclei, we produced and imaged two sets of samples. On the one hand, we used *Platynereis* heads to correlate the expression of the circadian clock gene *period* with the photoreceptor protein L-Cryptochrome (L-Cry) and the proliferation marker EdU (Fig. 2). On the other hand, we assessed expression of the posterior stem cell marker *hox3* with stabilized microtubules and EdU in regenerating tail samples (Fig. 3). In both cases, background staining in the respective channels was controlled by the use of HCR probes directed against an unrelated sponge gene (see Table 1 for probe targets, Table 2 for target proteins, and Supplementary File 2 for HCR probe sequences). Moreover, obtained patterns were compared against the result of published expression studies to validate the accuracy of our method.

### Covisualization of L-Cry, *period* mRNA, and EdU in heads

One of the interesting aspects about *P.dumerilii* is its use as a functional molecular model system for chronobiological analyses (Raible and Tessmar-Raible, 2014; Özpolat et al. 2021). At least two endogenous timing systems—a ∼24hr (plastic circadian-circalunidian) and a monthly (circalunar) one—coexist in *Platynereis* and are influenced by ambient light conditions (Zantke et al. 2013) (Poehn et al. 2022; Zurl et al. 2022). *Period (Pladu_per)* is a key gene of the circadian clock (Glossop and Hardin, 2002). The *Platynereis period* ortholog has previously been shown to be expressed in the head using riboprobe-based whole-mount ISH (Zantke et al. 2013). In accordance with the published pattern, HCR probes designed against *Platynereis period* demarcate cells in the posterior medial brain as well as in the eyes (arrows and asterisks, respectively, in Fig. 2A and D). In addition to this established pattern, we noted weaker, more ubiquitous *period* staining, which cannot be seen in the negative control (cf. arrowheads in Fig. 4B/B’). Whereas, this expression is not seen in the conventional ISH for *period*, it might be attributed to the higher sensitivity of HCR and its ability to detect single mRNA molecules (Choi et al.^2018^).

In order to coassess cell proliferation in the head during the night, we incubated worms in EdU solution from darkness onset to 2 hr before the lights turned (i.e., for a total of 6 hr). Visualization of EdU using click chemistry revealed positive cells in the anterior part of the head, in a dispersed, salt-and-pepper pattern with few cells also localizing to the posterior of the head (Fig. 2B and D). At the investigated stage, we did not observe many proliferating cells in the eyes, which exhibit continuous growth and noticeably increase in size before sexual maturation (Fischer et al. 2010; Pende et al. 2020; Özpolat et al. 2021a).

To test the compatibility of SHInE with IHC, immunostainings were performed on the same samples using antibodies raised against *Platynereis* L-Cry. L-Cry is a photoreceptor that shares regions of expression with *period* and is well described also on the sub-cellular level for *P.dumerilii* (Poehn et al. 2022; Zurl et al. 2022). In line with the published expression patterns, the acquisition of SHInE-processed heads revealed expression of L-Cry in the medial brain nuclei and in the eyes (arrows and asterisks, respectively, in Fig. 2C and D), in the same region as *period* RNA (Fig. 2A and D).

### Covisualization of acetylated alpha-tubulin, *hox3* mRNA, and EdU in posterior blastemas

Regenerating tissue after caudal amputation has been used in *Platynereis* for various studies of cell identity, regeneration and the hormonal control of maturation (Rosa et al. 2005; Balavoine, 2015; Starunov et al. 2015; Özpolat and Bely, 2016; Planques et al. 2018; Özpolat et al. 2021). A key gene involved in both posterior growth as well as regeneration is the homeobox gene *hox3*. It is expressed in a distinct set of cells believed to be ectoteloblasts (ectodermal stem cells). These cells are found in a region of high proliferation and expression of genes belonging to the germline multipotency program, called the segment addition zone (Gazave et al. 2013; Planques et al. 2018). We chose this gene for testing our protocol as it has a highly distinct, well-studied expression pattern. Cells positive for *hox3* also exhibit enlarged nucleoli, which makes it possible to identify them and validate the *hox3* staining.

Detection of *hox3* using HCR probes revealed a distinct region along the border between the pygidium and the regenerating segments (arrowheads in Fig. 3A and D). We colabelled samples using antiacetylated tubulin antibodies, which are established to visualize neurites of the nervous system. Antibody signal was found along the ventral nerve cord (VNC) and extended laterally into the animal's parapodia and posteriorly into the newly regenerated anal cirri (asterisk and arrowheads, respectively, in Fig. 3C and D). Both *hox3* HCR and antiacetylated tubulin labeling match the established patterns, demonstrating the compatibility of these two techniques with the SHInE protocol.

As with head samples, we also performed an additional labeling of proliferative cells by pretreating animals with EdU for 30 min just before fixation, and visualized EdU incorporated in cells undergoing S-phase using click chemistry. The pattern of proliferation is broad and matches previous observations of regenerating tissue after posterior amputation (Fig. 3B and D) (Planques et al. 2018). Taken together, the analyses performed in both head and posterior regenerates show that SHInE allows for codetection of nuclear EdU with both mRNA and protein.

### SHInE is compatible with tissue clearing

We have previously presented a novel method on tissue clearing and depigmentation named DEEP-Clear. This method is compatible with various labeling techniques, including IHC, fluorescent proteins, and EdU-labeling. Enhanced transparency and refractive index matching allow for high-resolution imaging of specimens up to the centimeter range (Pende et al. 2020). While DEEP-Clear was shown to allow for the detection of RNA using conventional riboprobes, the employed chemistry includes an alkaline solution, in which RNA is expected to be degraded over time. As *in situ* HCR only requires binding to short pieces of RNA, rather than alignment with longer mRNA molecules, we reasoned that after clearing, *in situ* HCR should yield comparable or even superior results when compared to riboprobe-based ISH. As a suitable test case for assessing the compatibility of SHInE with tissue clearing, we focused on the aforementioned expression domains of *Pladu-period* in the eyes and the oval-shaped domain (Fig. 4 B/B’) of the posterior medial forebrain. Whereas, worm eyes are strongly pigmented, and the brain is opaque, DEEP-Clear had been shown to improve visualization of signals in both tissues (Pende et al. 2020).

Indeed, a comparison of untreated samples (Fig. 4B’) and samples pre-processed using DEEP-Clear (Fig. 4B) revealed that tissue clearing was not only compatible with *in situ* HCR, but also led to a noticeable improvement of the signal. To quantify this effect, we compared *Pladu-period* signal in the medial brain with equivalent regions of samples hybridized with the unrelated sponge HCR probes (see Materials and methods section) carrying the same amplifier (Fig. 4A/A’). This quantification revealed a significant increase in signal-to-noise ratios upon tissue clearing (see Supplementary Fig. S1).

The degree to which tissue clearing improves signal might depend on the type of fluorophore used: When we investigated the expression of the PAR-domain protein 1 gene *(pdp1)*—that is expressed in a similar pattern—using a spectrally different HCR amplifier (B1 amplifier coupled to Alexa 546), tissue clearing did not improve HCR signal intensity to the same extent than it did for the B2 amplifier coupled to Alexa 647) (see Supplementary Fig. S2). For blastemal tissue that is neither pigmented nor particularly opaque, clearing did not improve the HCR signal noticably (Fig. 4C–D’).

In summary, we find that SHInE is compatible with tissue clearing using DEEP-Clear, which, depending on the type of tissue and the fluorophore used for labeling, can improve the HCR signal quality and reduce background signal.

## Discussion

Here we present a novel protocol that combines *in situ* HCR with IHC and EdU labeling of proliferating cells. The simultaneous visualization of RNA and proteins in the same tissue, as well as the high sensitivity of HCR-mediated RNA visualization, make the protocol suitable for detailed studies on the level of organisms, tissues, and cells.

As we demonstrate, it is possible to successfully apply SHInE using home-made reagents, with HCR probes designed with a free webtool, home-made buffers and custom antibodies. This serves to keep the protocol affordable and flexible to many biological questions. The combination of multiple labeling methods within the same steps also greatly reduces the time required compared to similar protocols, further increasing the accessibility of these kinds of experiments, or allowing researchers to sample more replicates or experimental conditions.

Additionally, SHInE gives the researcher full control over reagents. This opens the protocol up for future improvement or adaptation to other models and tissues. For example, dextran sulfate, which is part of the probe amplification buffer, has been reported to negatively impact some antibodies during IHC (Callahan et al. 1991), so changing the concentration of the reagent could help in such cases. At several stages of the protocol, we tested different HCR probe concentrations and amplification lengths and report our findings and suggestions in the main protocol (Supplementary File 1), providing a foundation for other researchers to adapt and modify the protocol to their requirements.

As we show, compatibility of SHInE with tissue clearing allows for improved HCR signal intensity in originally opaque and pigmented tissue. Not only do we provide evidence for the compatibility of DEEP-Clear with *in situ* HCR, but furthermore show an improvement in the signal-to-noise ratio of acquired images. However, the extent of this improvement may vary depending on tissue type and spectral range of the used fluorophores.

We believe that this protocol, with its flexibility and ease of use, combined with low cost and customization opportunities, will be a valuable resource. Whereas, we have focused on *Platynereis* as a model species, the broad applicability of *in situ* HCR and DEEP-Clear for various invertebrate and vertebrate species suggests that the combined protocol we present here can easily be adapted for other model systems, and thereby help to complement the powerful possibilities opened by scRNAseq in a variety of model species.

## Supplementary Material

icad007_Supplemental_Files

## Data Availability

All data relevant to this study are either included or referenced to within the main manuscript or its supplemental material.

## Notes

From the symposium “Neuroethology in the age of gene editing: New tools and novel insights into the molecular and neural basis of behavior" presented at the annual meeting of the Society for Integrative and Comparative Biology virtual annual meeting, January 3–February 28, 2023.

## References

[bib1] Albayrak C , Jordi CA, Zechner C, Lin J, Bichsel CA, Khammash M, Tay S. 2016. Digital quantification of proteins and mRNA in single mammalian cells. Mol Cell. 61:914–24. 26990994 10.1016/j.molcel.2016.02.030

[bib2] Asadulina A , Panzera A, Verasztó C, Liebig C, Jékely G. 2012. Whole-body gene expression pattern registration in *Platynereis* larvae. EvoDevo. 3:27. 23199348 10.1186/2041-9139-3-27 PMC3586958

[bib3] Balavoine G. 2015. Segment formation in Annelids: patterns, processes and evolution. Int J Dev Biol. 58:469–83. 10.1387/ijdb.140148gb 25690963

[bib4] Callahan LN , Phelan M, Mallinson M, Norcross MA. 1991. Dextran sulfate blocks antibody binding to the principal neutralizing domain of human immunodeficiency virus type 1 without interfering with gpl20-CD4 interactions. J Virol. 65:1543–50. 1995952 10.1128/jvi.65.3.1543-1550.1991 PMC239935

[bib5] Choi HMT , Calvert CR, Husain N, Huss D, Barsi JC, Deverman BE, Hunter RC, Kato M, Lee SM, Abelin ACT et al. 2016. Mapping a multiplexed zoo of mRNA expression. Development. 143:3632–7. 27702788 10.1242/dev.140137 PMC5087610

[bib6] Choi HMT , Schwarzkopf M, Fornace ME, Acharya A, Artavanis G, Stegmaier J, Cunha A, Pierce NA. 2018. Third-generation *in situ*hybridization chain reaction: multiplexed, quantitative, sensitive, versatile, robust. Development. 145:dev165753. 29945988 10.1242/dev.165753 PMC6031405

[bib7] Darmanis S , Gallant CJ, Marinescu VD, Niklasson M, Segerman A, Flamourakis G, Fredriksson S, Assarsson E, Lundberg M, Nelander S et al. 2015. Simultaneous multiplexed measurement of RNA and proteins in single cells. Cell Rep. 14:380–9. 26748716 10.1016/j.celrep.2015.12.021 PMC4713867

[bib8] Elagoz AM , Styfhals R, Maccuro S, Masin L, Moons L, Seuntjens E. 2022. Optimization of whole Mount RNA multiplexed *in situ&nbsp;*hybridization chain reaction with immunohistochemistry, clearing and imaging to visualize octopus neurogenesis. Front Physiol. 13: 882413. 10.1101/2022.02.24.481749 35711315 PMC9196907

[bib9] Fischer AH , Henrich T, Arendt D. 2010. The normal development of *Platynereis dumerilii* (Nereididae, Annelida). Front Zool. 7:31. 21192805 10.1186/1742-9994-7-31 PMC3027123

[bib10] da Fonseca R R , Albrechtsen A, Themudo GE, Ramos-Madrigal J, Sibbesen JA, Maretty L, Zepeda-Mendoza ML, Campos PF, Heller R, Pereira RJ. 2016. Next-generation biology: sequencing and data analysis approaches for non-model organisms. Mar Genom. 30:3–13. 10.1016/j.margen.2016.04.012 27184710

[bib11] Gazave E , Béhague J, Laplane L, Guillou A, Préau L, Demilly A, Balavoine G, Vervoort M. 2013. Posterior elongation in the annelid *Platynereis dumerilii* involves stem cells molecularly related to primordial germ cells. Dev Biol. 382:246–67. 23891818 10.1016/j.ydbio.2013.07.013

[bib12] Glossop NRJ , Hardin PE. 2002. Central and peripheral circadian oscillator mechanisms in flies and mammals. J Cell Sci. 115:3369–77. 12154068 10.1242/jcs.115.17.3369

[bib13] Hasse C , Rebscher N, Reiher W, Sobjinski K, Moerschel E, Beck L, Tessmar-Raible K, Arendt D, Hassel M. 2010. Three consecutive generations of nephridia occur during development of *Platynereis dumerilii* (Annelida, Polychaeta). Dev Dyn. 239:1967–76. 20549733 10.1002/dvdy.22331

[bib14] Hauenschild C , Fischer A. 1969. *Platynereis dumerilii*: Mikroskopische Anatomie, Fortpflanzung, Entwicklung. Großes Zoologisches Praktikum Heft 10b. 1–55.. Gustav Fischer Verlag. Stuttgart.

[bib15] Ibarra-García-Padilla R , Howard AGA, Singleton EW, Uribe RA. 2021. A protocol for whole-mount immuno-coupled hybridization chain reaction (WICHCR) in zebrafish embryos and larvae. Star Protoc. 2:100709. 34401776 10.1016/j.xpro.2021.100709 PMC8348268

[bib16] Kuehn E , Clausen DS, Null RW, Metzger BM, Willis AD, Özpolat BD. 2022. Segment number threshold determines juvenile onset of germline cluster expansion in *Platynereis dumerilii*. J Exp Zoology Part B Mol Dev Evol. 338:225–40. 10.1002/jez.b.23100 PMC9114164 34793615

[bib17] Kuehn E , Stockinger AW, Girard J, Raible F, Özpolat BD. 2019. A scalable culturing system for the marine annelid *Platynereis dumerilii*. PLoS One. 14:e0226156. 31805142 10.1371/journal.pone.0226156 PMC6894799

[bib18] Özpolat BD , Bely AE. 2016. Developmental and molecular biology of annelid regeneration: a comparative review of recent studies. Curr Opin Genet Dev. 40:144–53. 27505269 10.1016/j.gde.2016.07.010

[bib19] Özpolat BD , Randel N, Williams EA, Bezares-Calderón LA, Andreatta G, Balavoine G, Bertucci PY, Ferrier DEK, Gambi MC, Gazave E et al. 2021. The Nereid on the rise: *P latynereis* as a model system. EvoDevo. 12:10. 34579780 10.1186/s13227-021-00180-3 PMC8477482

[bib20] Pende M , Vadiwala K, Schmidbaur H, Stockinger AW, Murawala P, Saghafi S, Dekens MPS, Becker K, Revilla-i-Domingo R, Papadopoulos S-C et al. 2020. A versatile depigmentation, clearing, and labeling method for exploring nervous system diversity. Sci Adv. 6:eaba0365. 32523996 10.1126/sciadv.aba0365 PMC7259959

[bib21] Planques A , Malem J, Parapar J, Vervoort M, Gazave E. 2018. Morphological, cellular and molecular characterization of posterior regeneration in the marine annelid *Platynereis dumerilii*. Dev Biol. 445:189–210. 30445055 10.1016/j.ydbio.2018.11.004

[bib22] Poehn B , Krishnan S, Zurl M, Coric A, Rokvic D, Häfker NS, Jaenicke E, Arboleda E, Orel L, Raible F et al. 2022. A cryptochrome adopts distinct moon- and sunlight states and functions as sun- versus moonlight interpreter in monthly oscillator entrainment. Nat Commun. 13:5220. 36064778 10.1038/s41467-022-32562-z PMC9445029

[bib23] Raible F , Tessmar-Raible K. 2014. Platynereis dumerilii. Curr Biol. 24:R676–7. 25093553 10.1016/j.cub.2014.06.032

[bib24] Revilla-i-Domingo R , Rajan VBV, Waldherr M, Prohaczka G, Musset H, Orel L, Gerrard E, Smolka M, Stockinger A, Farlik M et al. 2021. Characterization of cephalic and non-cephalic sensory cell types provides insight into joint photo- and mechanoreceptor evolution. Elife. 10:e66144. 34350831 10.7554/eLife.66144 PMC8367381

[bib25] Rosa R , Prud'homme B, Balavoine G. 2005. caudal and even-skipped in the annelid *Platynereis dumerilii* and the ancestry of posterior growth. Evol Dev. 7:574–87. 16336411 10.1111/j.1525-142X.2005.05061.x

[bib26] Salic A , Mitchison TJ. 2008. A chemical method for fast and sensitive detection of DNA synthesis in vivo. Proc National Acad Sci. 105:2415–20. 10.1073/pnas.0712168105 PMC2268151 18272492

[bib27] Schindelin J , Arganda-Carreras I, Frise E, Kaynig V, Longair M, Pietzsch T, Preibisch S, Rueden C, Saalfeld S, Schmid B et al. 2012. Fiji: an open-source platform for biological-image analysis. Nat Methods. 9:676–82. 22743772 10.1038/nmeth.2019 PMC3855844

[bib28] Schwarzkopf M , Liu MC, Schulte SJ, Ives R, Husain N, Choi HMT, Pierce NA. 2021. Hybridization chain reaction enables a unified approach to multiplexed, quantitative, high-resolution immunohistochemistry and *in situ* hybridization. Dev Camb Engl. 148:dev199847. 10.1242/dev.199847 PMC8645210 35020875

[bib29] Stark R , Grzelak M, Hadfield J. 2019. RNA sequencing: the teenage years. Nat Rev Genet. 20:631–56. 31341269 10.1038/s41576-019-0150-2

[bib30] Starunov VV , Dray N, Belikova EV, Kerner P, Vervoort M, Balavoine G. 2015. A metameric origin for the annelid pygidium?. Bmc Evol Biol. 15:25. 25880037 10.1186/s12862-015-0299-z PMC4357181

[bib31] Tainaka K , Kuno A, Kubota SI, Murakami T, Ueda HR. 2015. Chemical principles in tissue clearing and staining protocols for whole-body cell profiling. Annu Rev Cell Dev Bi. 32:1–29. 10.1146/annurev-cellbio-111315-125001 27298088

[bib32] Tessmar-Raible K , Steinmetz PRH, Snyman H, Hassel M, Arendt D. 2005. Fluorescent two-color whole mount *in situ*hybridization in *Platynereis dumerilii* (Polychaeta, Annelida), an emerging marine molecular model for evolution and development. BioTechniques. 39:460–4. 16235555 10.2144/000112023

[bib33] Vieites-Prado A , Renier N. 2021. Tissue clearing and 3D imaging in developmental biology. Development. 148:dev199369. 34596666 10.1242/dev.199369 PMC8497915

[bib34] Zantke J , Ishikawa-Fujiwara T, Arboleda E, Lohs C, Schipany K, Hallay N, Straw AD, Todo T, Tessmar-Raible K. 2013. Circadian and circalunar clock interactions in a marine annelid. Cell Rep. 5:99–113.. 24075994 10.1016/j.celrep.2013.08.031 PMC3913041

[bib35] Zattara EE , Özpolat BD. 2020. Developmental biology of the sea urchin and other marine invertebrates. Methods Mol Biol. 2219:163–80. 10.1007/978-1-0716-0974-3_10 33074540

[bib36] Zurl M , Poehn B, Rieger D, Krishnan S, Rokvic D, Rajan VBV, Gerrard E, Schlichting M, Orel L, Ćorić A et al. 2022. Two light sensors decode moonlight versus sunlight to adjust a plastic circadian/circalunidian clock to moon phase. Proc National Acad Sci. 119:e2115725119. 10.1073/pnas.2115725119 PMC9295771 35622889

