# Supplementary material for: A Fast And Versatile Method for Simultaneous HCR, Immunohistochemistry And Edu Labeling (SHInE)
Source: Integr Comp Biol. 2023 Mar 2;63(2):372–81. doi: 10.1093/icb/icad007 (PMC10445416; doi:10.1093/icb/icad007)
Supplement: icad007_Supplemental_Files [file icad007_supplemental_files.zip › icb-2022-0168-File008.pdf]

A fast and versatile method for simultaneous HCR, immunohistochemistry and EdU labeling (SHInE)

Aida Ćorić, Alexander W. Stockinger, Petra Schaffer, Dunja Rokvić,
Kristin Tessmar-Raible and Florian Raible

Supplementary Figures S1, S2

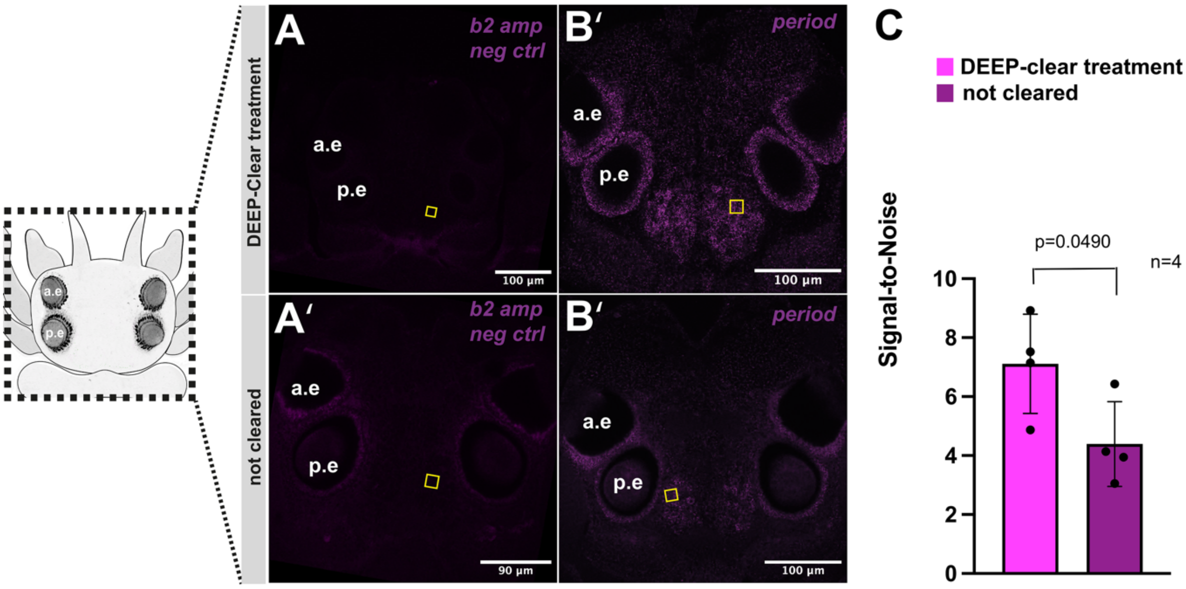

Supplementary Figure S1. Quantitative assessment of the effects of tissue clearing on HCR signal intensity using the Alexa-647-coupled B2 amplifier. (A-B') Panels corresponding to Fig. 4A-B'; Yellow boxes indicate areas in the posterior medial forebrain of treated and untreated specimens in which fluorescence was measured in these samples. **(C)** Signal-to-Noise ratios were calculated and compared for treated and untreated samples. Individual data points (n=4 biological replicates) and mean values with SD are shown. P-values of unpaired, two-tailed *t*-test, p=0.0490.

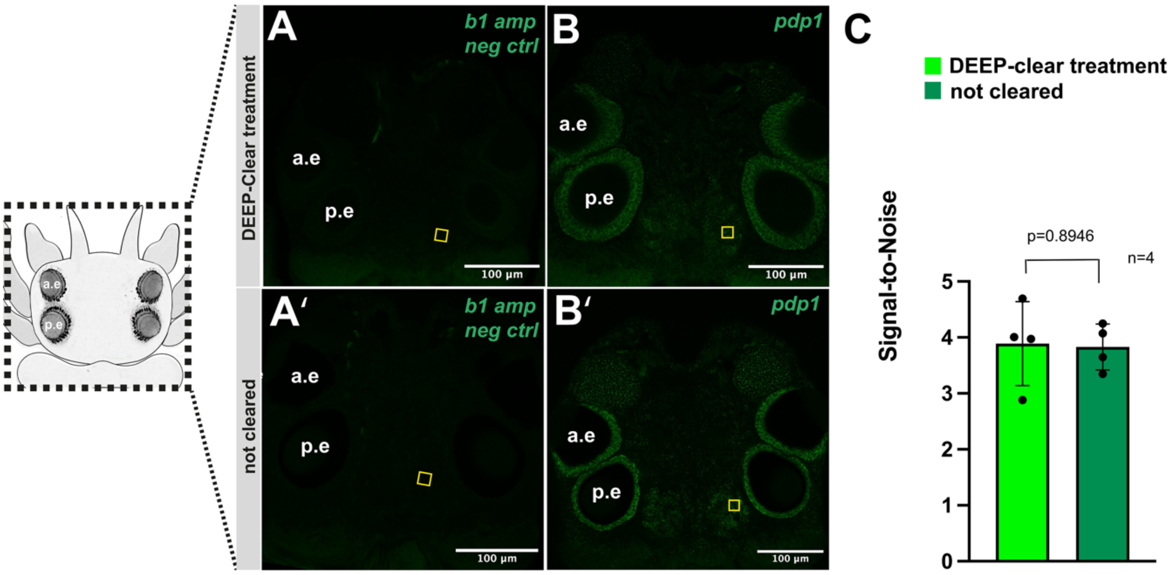

Supplementary Figure S2. Qualitative and quantitative assessment of the effects of tissue clearing on HCR signal intensity using the Alexa-546-coupled B1 amplifier. (A-B') As for Figure S1, panels show negative controls (A, A') that exhibit some autofluorescence around and in the eyes. **(B,B')** *Platynereis pdp1* – detected using the Alexa-546-coupled B1 amplifier – is found around the eyes and nuclei of posterior medial forebrain. Less centralized *Pladu-pdp1* is detected in both untreated and treated samples. Following tissue clearing, HCR signal is particularly strong around the eyes. For this detection, HCR signal seems more distinct and sharper in the untreated sample (B') compared to the cleared specimen (B). Yellow boxes in (A-B') indicate areas in which signal was quantified; **(C)** As in Figure S1, fluorescence intensity was measured in depicted areas of the posterior medial forebrain for treated and untreated samples and their negative controls (A-B'). Signal-to-Noise ratios were calculated and compared for treated and untreated samples. Individual data points (n=4 biological replicates) and mean values with SD are shown. P-values of unpaired, two-tailed *t*-test, p=0.8946.
